# Coexistence of Mycosis Fungoides and Hodgkin Lymphoma in a Pediatric Patient: A Report of a Rare Case

**DOI:** 10.7759/cureus.112474

**Published:** 2026-07-11

**Authors:** Cynthia Shanat Cruz Medina, José Alfredo Barrientos Hernandez, Victor Manuel Vargas Vargas, Miriam Ixell Escamilla López, Eduardo Tellez Bernal

**Affiliations:** 1 Pediatric Oncology, Unidad Médica Onco-Hematológica, Puebla, MEX; 2 Research, Unidad Médica Onco-Hematológica, Puebla, MEX; 3 Oncology, Unidad Médica Onco-Hematológica, Puebla, MEX

**Keywords:** dual malignancy, hodgkin lymphona, immunotherapy response, mycosis fungiodes, pd-l1 expression, pediatric case, pediatric hematology, pediatrics, primary cutaneus lymphoma

## Abstract

Mycosis fungoides (MF) is the most common primary cutaneous T‑cell lymphoma, usually affecting adults aged 50-60 years; pediatric presentation is rare. We report a 12‑year‑old patient whose course spanned five years; skin lesions began at age 8, were misdiagnosed and treated as inflammatory dermatosis with topical steroids for four years. At age 12, B‑symptoms, lymphadenopathy and organomegaly led to the diagnosis of MF Stage IA (T1bNxM0B0) and synchronous Hodgkin lymphoma (HL) Stage IVB, with an Epstein-Barr virus (EBV) viral load of 2.5 log₁₀ copies/mL. Treatment with three cycles of CHOP (cyclophosphamide, doxorubicin, vincristine, and prednisone) + pembrolizumab due to drug availability, followed by six cycles of PC‑AVBE (prednisone, cyclophosphamide, doxorubicin, vincristine, bleomycin, and etoposide) and radiotherapy, achieving complete remission. This case highlights diagnostic challenges, immune dysregulation, and adapted treatment in resource‑limited settings.

## Introduction

Mycosis fungoides (MF) is the most common subtype of cutaneous lymphoma; among these, the T‑cell phenotype predominates, accounting for approximately 50% of all primary cutaneous lymphomas [1]. It typically affects individuals between 50 and 60 years of age and shows a male predominance [2,3]. Notably, this kind of presentation is highly unusual; our patient was only 12 years old at diagnosis, far younger than the established age distribution.

Pathophysiologically, the origins of MF involve clonally transformed CD4+ T lymphocytes driven by shared immune dysregulation, chronic antigenic stimulation, impaired apoptotic signaling, and dysregulated immune surveillance, circumstances that may confer an increased predisposition to the development of secondary lymphoid neoplasms [1-3].

When confined to the skin, MF follows an indolent progression, evolving from patch‑stage lesions to infiltrated plaques and subsequently to tumor‑stage disease [4]. Prognosis is closely related to disease stage and progression: survival rates are approximately 97-98% for early‑stage disease, 83% for generalized cutaneous involvement, 42% for advanced stages with tumor formation, and decrease to around 20% in patients with lymph node involvement [2]. Patients with MF have a significantly elevated risk of developing hematological malignancies, including both Hodgkin lymphoma (HL) and non‑Hodgkin lymphoma (NHL) [3]. Although the pathophysiological mechanisms underlying this association are not fully understood, proposed explanations include alterations in hematopoietic stem cell biology, chronic antigenic stimulation, and persistent immune dysregulation linked to neoplastic T‑cell proliferation [4].

The most common presentation is the patch stage, followed by plaque‑stage disease, in which patients exhibit long‑standing, scaly macules that are generally asymptomatic and predominantly located on the trunk and sun‑protected areas [5]. Early‑stage disease is often clinically silent, although pruritus may occur and can become severe in advanced phases - particularly with deep lesions, erythrodermic MF, or Sézary syndrome - presentations that may mimic adult‑onset atopic dermatitis [6].

In the early stages, the lack of significant symptoms frequently leads patients to disregard the condition. Furthermore, it may be misdiagnosed both clinically and histopathologically, being confused with papulosquamous or eczematous disorders such as psoriasis, parapsoriasis, pityriasis rubra pilaris, chronic actinic dermatitis, or asteatotic dermatitis [7]. Most patients require multiple biopsies over several years before a definitive diagnosis is established. The average time from symptom onset to diagnosis ranges from three to four years, although in some cases it may extend for decades [8].

Against this background, we present the case of a pediatric patient diagnosed with MF who developed synchronous HL, highlighting the rarity of this association, the diagnostic challenges encountered, and the pathophysiological and therapeutic implications arising from the coexistence of both entities.

## Case presentation

A 12‑year‑old male patient, with no relevant personal or family history, was evaluated for a dermatological condition evolving over four years. The initial presentation, four years prior, included alopecia areata, crusted and erythematous scaly plaques, non‑pruritic in nature, and areas of hypopigmentation; lesions were initially confined to the upper extremities and dorsum of the hands, later becoming generalized to the trunk. He received intermittent topical treatment with hydrocortisone and tacrolimus 0.1%, achieving partial remission but with frequent recurrences.

One year before the current evaluation, cutaneous progression was noted, characterized by the appearance of nodular and erythrodermic lesions extending to the trunk and lower extremities, as well as cervical and inguinal lymphadenopathy - intermittent, painless, and measuring up to 2 cm in diameter (Figures 1, 2). Seven months prior, lymphadenopathy became persistent and progressively enlarged, exceeding 2 cm in diameter in cervical chains (levels IIa/b, IIIa/b), reaching 3 cm in the left axilla, and measuring > 2 cm in inguinal regions.

**Figure 1:**
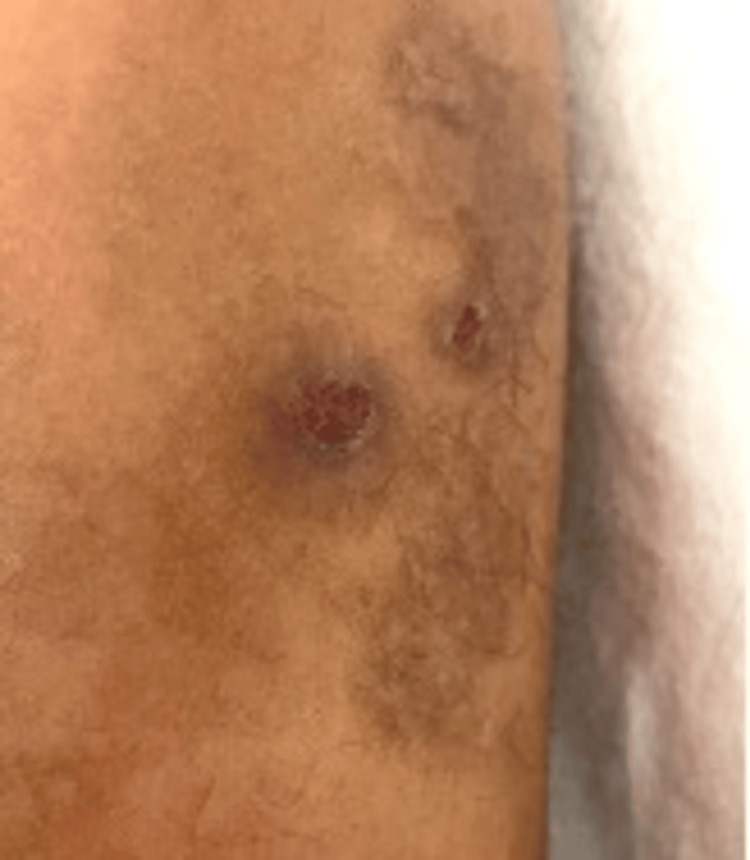
Erythematous and violaceous infiltrated plaques with ulcerated and crusted surfaces, distributed on the upper limb.

**Figure 2:**
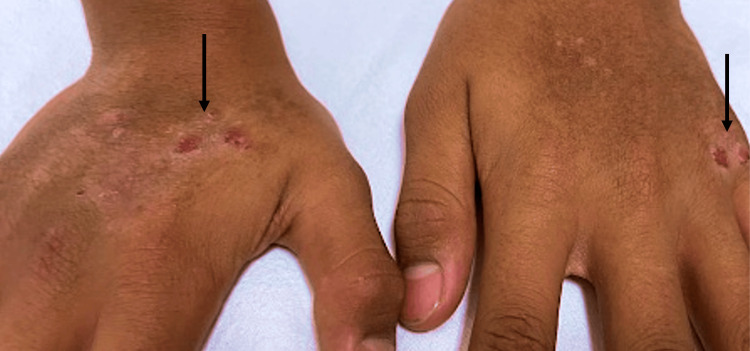
Bilateral involvement of hands showing erythematous patches, hypopigmented macules, and small nodules. Black arrows show multiple nodules and erythematous patches.

Physical examination revealed hepatomegaly of 3 cm below the right costal margin and splenomegaly of 3 cm below the left costal margin. Given the suspicion of a lymphoproliferative disorder, fluorine-18 fluorodeoxyglucose (¹⁸F‑FDG) positron emission tomography-computed tomography (PET‑CT) was performed, demonstrating increased and generalized metabolic activity in cervical, axillary, mediastinal, retroperitoneal, and inguinal lymph nodes, as well as diffuse uptake in the liver and spleen (Figure 3). The differential diagnosis included chronic inflammatory dermatoses (atopic eczema, psoriasis, pityriasis alba), other primary cutaneous T‑cell lymphomas, systemic lymphomas, and infectious causes such as Epstein‑Barr virus mononucleosis and tuberculosis. Final distinction was achieved by integrating clinical evolution, histopathology, immunohistochemistry, and clonality studies.

**Figure 3:**
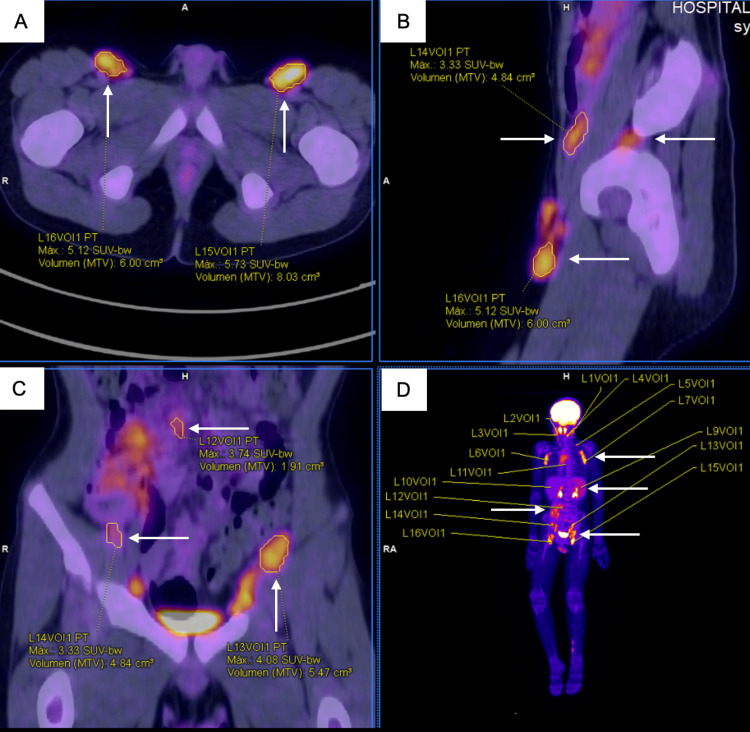
Fluorine-18 fluorodeoxyglucose (¹⁸F-FDG) positron emission tomography-computed tomography (PET-CT) scan. (A) Axial view: Bilateral inguinal lymph nodes showing intense metabolic activity (SUVmax 5.12–5.73); (B) Sagittal view: Abnormal uptake in iliac and inguinal regions, confirming nodal involvement; (C) Coronal view: Pathological activity in pelvic and abdominal lymph nodes (SUVmax 3.33–4.08); (D) Whole body view: Generalized lymphadenopathy affecting cervical, axillary, mediastinal, retroperitoneal, and inguinal chains, with additional uptake in the liver and spleen; consistent with disseminated disease. White arrows indicate areas of increased metabolic activity. SUVmax: maximum standardized uptake values.

Serial skin biopsies showed epidermal lymphocytic infiltration with epidermotropism, findings consistent with MF (Figure 4).

**Figure 4:**
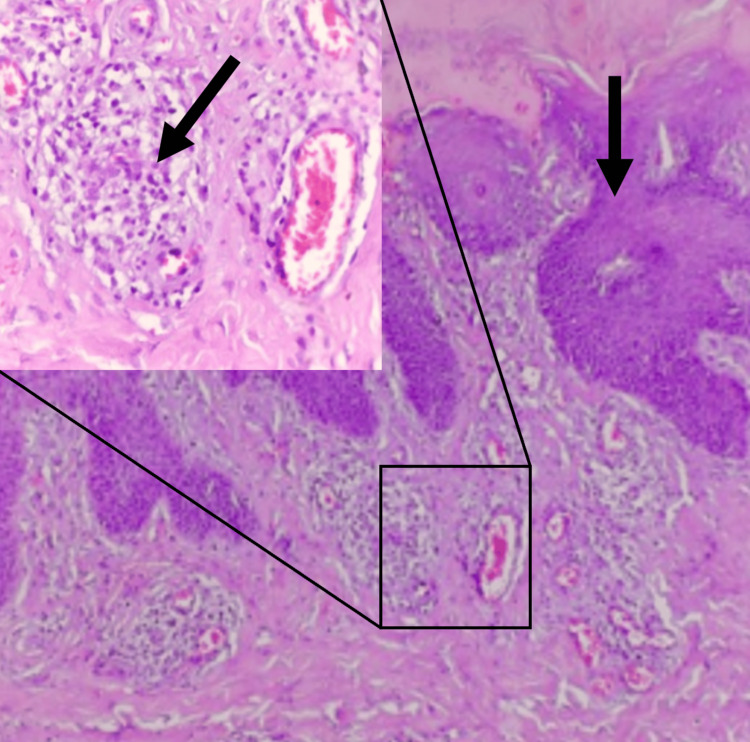
Histological features of mycosis fungoides. Epidermal acanthosis (arrow in the main panel), hyperkeratosis, and spongiosis are observed, along with intraepithelial lymphocytosis and dermal infiltration (arrow in the inset); findings confirm cutaneous T‑cell lymphoma.

Subsequently, lymph node biopsy revealed Reed‑Sternberg cells positive for CD30 and CD15 (Figure 5), confirming the diagnosis of classic HL.

**Figure 5:**
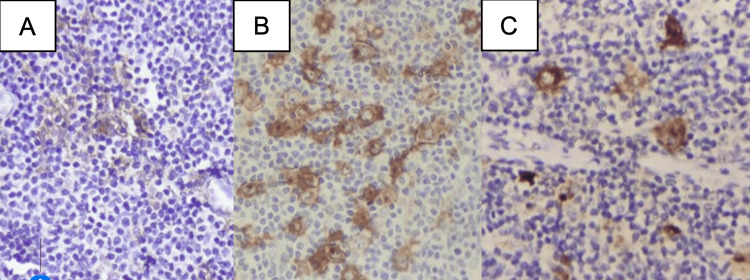
Immunohistochemical staining. (A) PD-L1: Positive expression (CSP score 5); (B) CD30: Strong membranous positivity in neoplastic cells; (C) CD15: Positive reaction in membrane and Golgi zone - findings confirm classic Hodgkin lymphoma. CSP: combined positive score, PD‑L1: programmed death‑ligand 1, CD: cluster of differentiation.

The clinical course spanned approximately five years; skin lesions consistent with early MF began at age 8 and were misdiagnosed and treated as inflammatory dermatosis with topical corticosteroids for four years. At age 12, B‑symptoms, generalized lymphadenopathy and organomegaly led to the diagnosis of MF and synchronous classic HL.

MF was staged according to the International Society for Cutaneous Lymphomas/European Organization of Research and Treatment of Cancer (ISCL/EORTC) TNMB (tumor, node, metastasis, blood) classification as stage IA (T1bNxM0B0). In parallel, classical HL was staged according to the Lugano classification as stage IVB due to lymph node involvement above and below the diaphragm, hepatic and splenic infiltration, and the presence of B symptoms. Epstein-Barr virus viral load was determined by quantitative PCR, revealing a positive result of 2.5 log₁₀ copies/mL.

The patient received systemic therapy consisting of chemotherapy and immunotherapy. Initial treatment included three cycles of the CHOP regimen (cyclophosphamide, doxorubicin, vincristine, and prednisone) plus pembrolizumab because of institutional logistical constraints and limited drug availability. Once drug availability was secured and following a partial clinical response, first-line treatment was switched to the PC-AVBE (prednisone, cyclophosphamide, doxorubicin, vincristine, bleomycin, and etoposide) regimen, which was administered for six cycles, followed by involved‑field radiotherapy to cervical and axillary regions at a total dose of 24 Gy with no notable side effects. This strategy resulted in a complete response, with total resolution of cutaneous lesions (Figure 6), lymphadenopathy, and organomegaly, confirmed by follow‑up PET‑CT (Figure 7). Currently, the patient remains under active surveillance with no evidence of recurrence.

**Figure 6:**
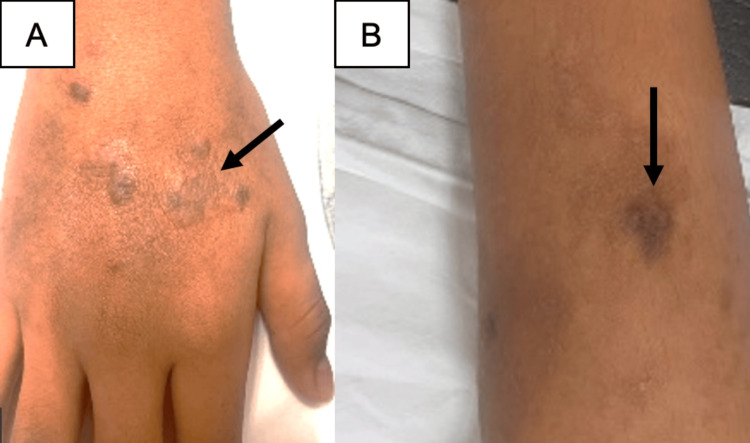
Complete clinical remission after treatment. Full resolution of infiltrated plaques and nodules in the left hand (A) and in the left upper limb (B), with only faint residual hyperpigmentation. Black arrows show residual hyperpigmentation of previous lesions.

**Figure 7:**
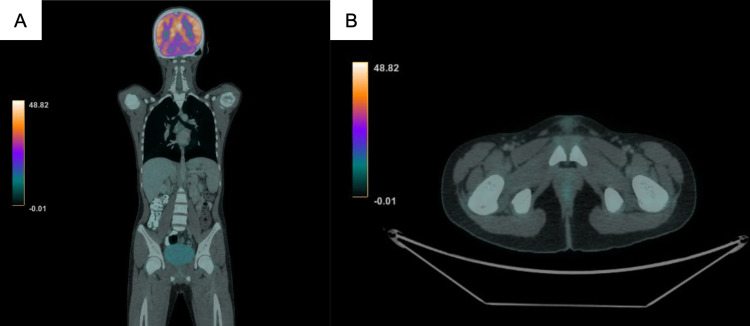
Follow-up positron emission tomography-computed tomography (PET CT) scan. (A) Coronal whole‑body projection showing only physiological distribution of the radiotracer. (B) Axial pelvic view showing no abnormal hypermetabolic zones. No pathological uptake is detected in lymph nodes, skin, liver, spleen, or other organs, confirming complete remission with no evidence of active disease.

## Discussion

Mycosis fungoides (MF) is the most common primary cutaneous T‑cell lymphoma, characterized by an indolent clinical course and prolonged evolution [1,4]. In the pediatric population, its low incidence and clinical similarity to benign dermatoses contribute to significant diagnostic delays [7,8].

MF exhibits variable morphology, leading to classification into clinical variants: classic, hypopigmented, erythrodermic, and folliculotropic, among others [9]. The typical presentation begins with patches that may progress to plaques and tumors; however, the hypopigmented variant predominates in younger patients and is associated with a more favorable prognosis [5]. Histologically, the diagnosis relies on identifying characteristic epidermal infiltration and the formation of intraepidermal collections of neoplastic lymphocytes, known as Pautrier microabscesses [10].

Classic Hodgkin lymphoma is a monoclonal B‑cell neoplasia, characterized by the presence of neoplastic cells within a microenvironment rich in inflammatory cells. Biologically, these two entities arise from distinct cell lineages (T‑cells and B‑cells, respectively); nevertheless, shared pathogenetic mechanisms have been described, including alterations in the tumor microenvironment and clonal relationships between lymphoid neoplasms [11].

The coexistence of MF and HL is exceptionally rare, with fewer than a dozen cases reported in the literature, predominantly in adult patients. Most publications describe a metachronous presentation, where MF precedes the development of HL by several years. Concurrent or associated presentations are extremely uncommon and, notably, this association had not been previously reported to the best of our knowledge in the pediatric age group.

It has been hypothesized that prolonged cutaneous treatment with immunosuppressive agents in patients with MF may alter the immune microenvironment, thereby favoring the development of HL. In this scenario, chronic inflammation creates an immune‑dysregulated microenvironment where early mycosis fungoides shows nearly identical clinical and histological features to reactive atopic dermatitis, frequently resulting in years of misdiagnosis and prolonged topical steroid use; persistent antigenic stimulation, cumulative genetic damage in skin‑homing CD4+ T cells and impaired local antitumor surveillance are recognized mechanisms driving malignant transformation and an increased risk of second lymphoid neoplasms such as Hodgkin lymphoma [3]. In the study by Goyal et al. (2020), 14 patients diagnosed with MF who subsequently developed HL were identified in the Surveillance, Epidemiology, and End Results (SEER) database, representing an incidence of 2/1,000, compared with 0.027/1,000 in the general population [3]. In that series, 71.4% were male, the mean age at diagnosis of MF was 55.5 years (minimum reported age 34 years), the mean age at HL diagnosis was 61 years, and the average latency between the two diagnoses was three years.

Given the indolent nature and low frequency of MF in the pediatric population, disease progression occurs over several years, which directly impacts overall survival. However, prognosis may worsen if patients receive incorrect diagnoses or inadequate therapies [12].

Further research is required to improve diagnostic tools for MF. Ongoing studies utilizing gene expression profiling and transcriptomic analysis aim to enable early detection and better characterize the relationship between MF and other lymphoproliferative neoplasms [13].

Therapeutic management was particularly challenging because no standardized first‑line regimen exists for lymphomas comprising Mycosis fungoides and synchronous classic Hodgkin lymphoma, given their rarity. The institutional standard of care for stage IVB pediatric Hodgkin lymphoma is PC‑AVBE, which was therefore the intended treatment from diagnosis; however, CHOP combined with pembrolizumab was selected temporarily as the best available evidence‑based alternative, limited to three cycles, entirely due to drug supply disruptions, logistical barriers and institutional dispensing policies in force at that moment, and never as a preferred option [14].

Treatment with CHOP plus pembrolizumab resulted in partial remission, confirming clinical activity in this setting. This favorable outcome supports immune checkpoint inhibition as a viable therapeutic alternative in carefully selected patients, in keeping with the PD‑L1 expression detected in our samples.

To date, to the best of our knowledge, approximately seven cases have been documented in the literature describing patients with mycosis fungoides or Sézary syndrome who developed Hodgkin lymphoma either synchronously or metachronously (Table 1). This scarcity highlights the rarity of the association and underscores a significant gap in the understanding of the underlying pathophysiological mechanisms, including the potential role of shared clonal alterations, chronic immune dysfunction, and iatrogenic factors.

**Table 1:** Reported cases of mycosis fungoides with synchronous or metachronous occurrence of Hodgkin lymphoma.

Reference (year)	Primary diagnosis	Associated malignancy	n	Age (years)
Zackheim et al. (2002) [15]	Mycosis fungoides	Hodgkin lymphoma	1 / 69	<20
Park et al. (1991) [16]	Mycosis fungoides	Hodgkin lymphoma	1	37
Kaufman et al. (1987) [17]	Mycosis fungoides	Hodgkin lymphoma	1	30
Zanelli et al. (2021) [11]	Mycosis fungoides	Hodgkin lymphoma	1	71
Geldenhuys et al. (1999) [18]	Mycosis fungoides	Hodgkin lymphoma	1	63
Chen et al. (2020) [19]	Mycosis fungoides	Hodgkin lymphoma	1	63
Davis et al. (1992) [20]	Sézary syndrome	Hodgkin lymphoma	1	31
Present case	Mycosis fungoides	Hodgkin lymphoma	1	12

## Conclusions

We report one of the first documented cases of concomitant mycosis fungoides and Hodgkin lymphoma in a pediatric patient. In this patient, the prolonged course and polymorphic presentation of lesions reflect the well‑known diagnostic challenges of mycosis fungoides, while systemic involvement allowed early identification of the second neoplasm. Treatment with CHOP plus pembrolizumab resulted in partial remission, confirming clinical activity in this setting. This favorable outcome supports the use of immune checkpoint inhibition as a valid therapeutic option in selected patients, consistent with the PD‑L1 expression detected in our case. This case raises important questions regarding the pathophysiological mechanisms underlying the concurrence of these two diseases, as well as the precipitating factors in this age group. Close clinical surveillance and timely intervention are essential to optimize prognosis and survival in these patients.
